# Social Network Analysis Shows Direct Evidence for Social Transmission of Tool Use in Wild Chimpanzees

**DOI:** 10.1371/journal.pbio.1001960

**Published:** 2014-09-30

**Authors:** Catherine Hobaiter, Timothée Poisot, Klaus Zuberbühler, William Hoppitt, Thibaud Gruber

**Affiliations:** 1 School of Psychology and Neuroscience, University of St. Andrews, Fife, United Kingdom; 2 Budongo Conservation Field Station, Masindi, Uganda; 3 Département de Biologie, Chimie et Géographie, Université du Québec à Rimouski, Rimouski, Québec, Canada; 4 Québec Centre for Biodiversity Sciences, Montréal, Québec, Canada; 5 Department of Comparative Cognition, Institute of Biology, University of Neuchâtel, Neuchâtel, Switzerland; 6 Animal and Environment Research Group, Anglia Ruskin University, Cambridge, United Kingdom; Emory University, United States of America

## Abstract

Social network analysis methods have made it possible to test whether novel behaviors in animals spread through individual or social learning. To date, however, social network analysis of wild populations has been limited to static models that cannot precisely reflect the dynamics of learning, for instance, the impact of multiple observations across time. Here, we present a novel dynamic version of network analysis that is capable of capturing temporal aspects of acquisition—that is, how successive observations by an individual influence its acquisition of the novel behavior. We apply this model to studying the spread of two novel tool-use variants, “moss-sponging” and “leaf-sponge re-use,” in the Sonso chimpanzee community of Budongo Forest, Uganda. Chimpanzees are widely considered the most “cultural” of all animal species, with 39 behaviors suspected as socially acquired, most of them in the domain of tool-use. The cultural hypothesis is supported by experimental data from captive chimpanzees and a range of observational data. However, for wild groups, there is still no direct experimental evidence for social learning, nor has there been any direct observation of social diffusion of behavioral innovations. Here, we tested both a static and a dynamic network model and found strong evidence that diffusion patterns of moss-sponging, but not leaf-sponge re-use, were significantly better explained by social than individual learning. The most conservative estimate of social transmission accounted for 85% of observed events, with an estimated 15-fold increase in learning rate for each time a novice observed an informed individual moss-sponging. We conclude that group-specific behavioral variants in wild chimpanzees can be socially learned, adding to the evidence that this prerequisite for culture originated in a common ancestor of great apes and humans, long before the advent of modern humans.

## Abstract 2

Network-based diffusion analysis demonstrates that a novel tool-use behavior, “moss-sponging”, spread via social learning in a wild East-African chimpanzee community.

## Author Summary

Chimpanzees are widely considered as the most “cultural” of all animals, despite the lack of direct evidence for the spread of novel behaviors through social learning in the wild. Here, we present a novel, dynamic network-based diffusion analysis to describe the acquisition patterns of novel tool-use behavior in the Sonso chimpanzee community of Budongo Forest, Uganda. We find strong evidence for social transmission of “moss-sponging” (the production of a sponge consisting of moss) along the innovators' social network, demonstrating that wild chimpanzees learn novel tool-use behaviors from each other and supporting the more general claim that some of the observed behavioral diversity in wild chimpanzees should be interpreted as “cultural.” Our model also estimated that, for each new observation, naïve individuals enhanced their chances of developing moss-sponging by a factor of 15. We conclude that group-specific behavioral variants can be socially learned in wild chimpanzees, addressing an important critique of the claim of culture in our closest relatives.

## Introduction

Progress in network analysis has made it possible to test whether the spread of novel behaviors in animal groups has occurred through individual learning or social transmission [1]–[5]. This method has been successfully applied in several species, including primates [3],[6],[7]. One particularly relevant example was the social spread of a novel foraging technique, lobtail feeding, in humpback whales (*Megaptera novaeangliae*), detected through Network-Based Diffusion Analysis (NBDA) [8]. The NBDA technique tests whether or not a novel behavior spreads along a social network, as would be expected if social transmission were involved [2],[8]. Although powerful, one important limitation of NBDA as it has been used so far in animal behavior studies is that it treats social networks as static. Static networks based directly on observations of the target behavior do not have a time dimension and so do not take into account the fact that an observation event can only influence the subsequent, and not the previous, rate of learning of the novel behavior (see Materials and Methods for an example). In contrast, if observation conditions allow for documentation of individuals that have witnessed specific events of the target behavior, then a dynamic network can be used. Dynamic networks change to reflect the time course of the observations and are therefore more powerful than static networks, by tracing which individuals are likely to have observed the novel behavior across time.

Here, we developed a novel version of NBDA that relies on instances of actual demonstrations of the novel behavior across time, rather than employing patterns of association as a proxy for demonstrations. We applied this method to two novel tool-use behaviors that appeared in the Sonso chimpanzee community of the Budongo Forest, Uganda (*Pan troglodytes schweinfurthii*). Our findings allowed us to directly address one persistent criticism faced by the hypothesis that chimpanzee behavioral diversity should be interpreted as cultural: the lack of direct evidence for social transmission of novel behaviors in the wild [9].

Claims of culture in animals are usually based on excluding genetic or ecological explanations for group-specific behavioral variation, the “exclusion method” [10]–[15]. Although widely used in animal behavior research, this method is vulnerable to counterarguments that seek to explain behavioral variation by genetic factors or with the local ecology [16]. Chimpanzees play a key role in this literature [10], with substantial indirect evidence for social transmission of behavioral innovations [17]–[21]. A good illustration is the presence or absence of nut-cracking in East and West African populations and in some neighbouring West African groups [22]. Studies in the wild have also found that the environment does play an important role in explaining some differences, notably by triggering behavioral innovation, the raw material for subsequent social transmission. However, environmental differences cannot account for all of the observed variation, suggesting a role for social learning processes [23]–[27]. In captivity, the evidence for social learning and transmission of novel behavior is undisputed, suggesting that the observed behavioral variation in the wild is an expression of culture in chimpanzees [28]–[34]. Nevertheless, skepticism has remained, as it is difficult to rule out an unmeasured ecological variable as the cause of observed group differences. In addition, to date, there have been no direct demonstrations of novel behavior spreading socially within a wild chimpanzee group, and it has remained unclear whether similar learning mechanisms are at work in humans and other animals, rendering it hard to draw evolutionary inferences on whether chimpanzee and human cultures may result from fundamentally similar or different acquisition processes [16],[35]–[37]. This ambiguity could be resolved by testing whether the spread of novel behavior follows the pathway predicted by social transmission.

**Figure 1:**
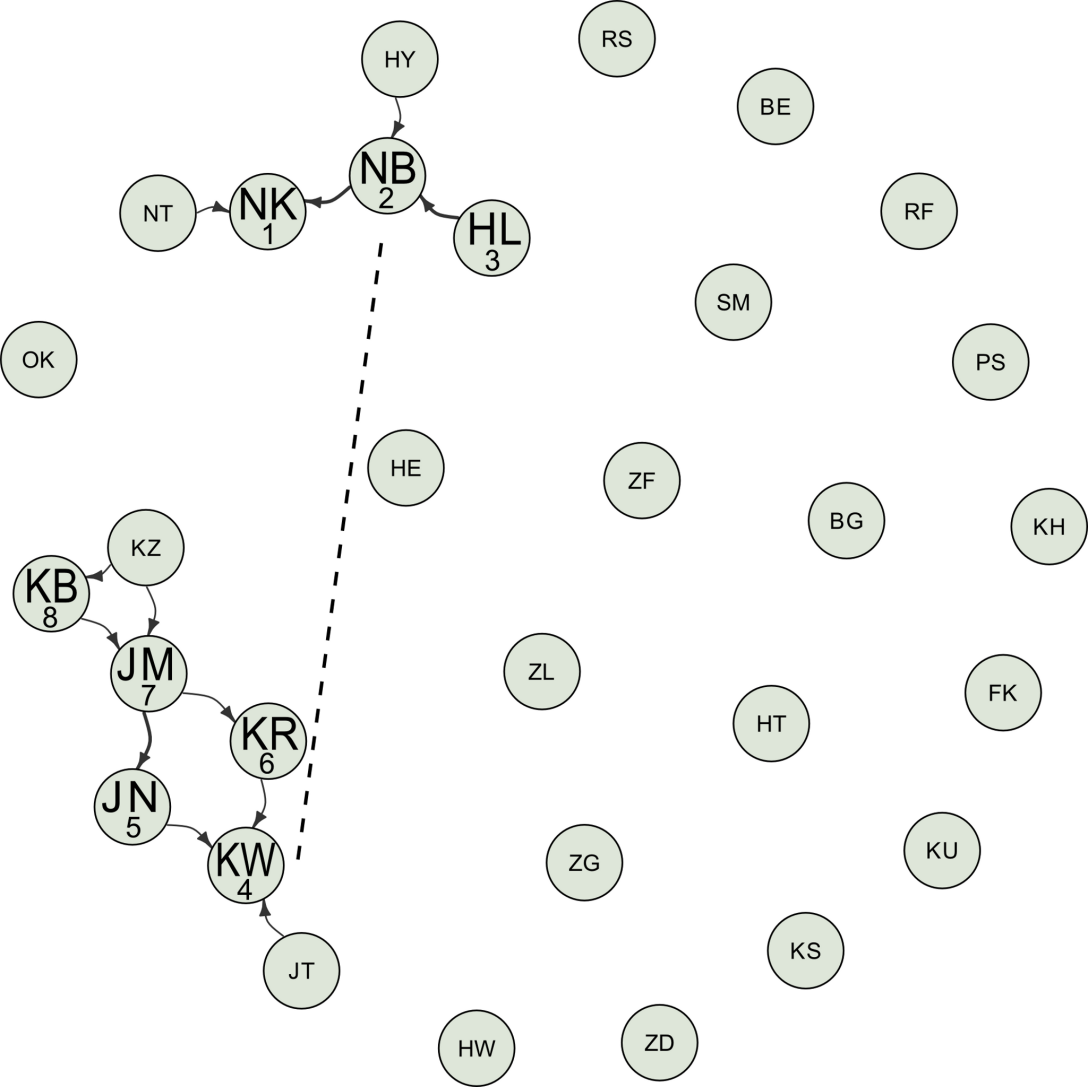
Visualization of the static interaction networks for the moss-sponging behavior for all 30 individuals. Graphs are laid out using the Fruchterman–Reingold weighted algorithm. Labels on the nodes indicate the identity of individuals (see Supporting Information). Individuals with large label size developed the behavior, whereas individuals with small label size did not. Numbers under the large label indicate the order of acquisition of the behavior. The width of the arrows linking individuals is proportional to the number of times an interaction event was recorded between any two individuals and represented according to the convention “X→Y” means that Y was observed by X. Dashed line indicates potential product-based social learning by individual KW who re-used a moss-sponge. Data were deposited in the Dryad repository: http://dx.doi.org/10.5061/dryad.m6s21.

**Figure 2:**
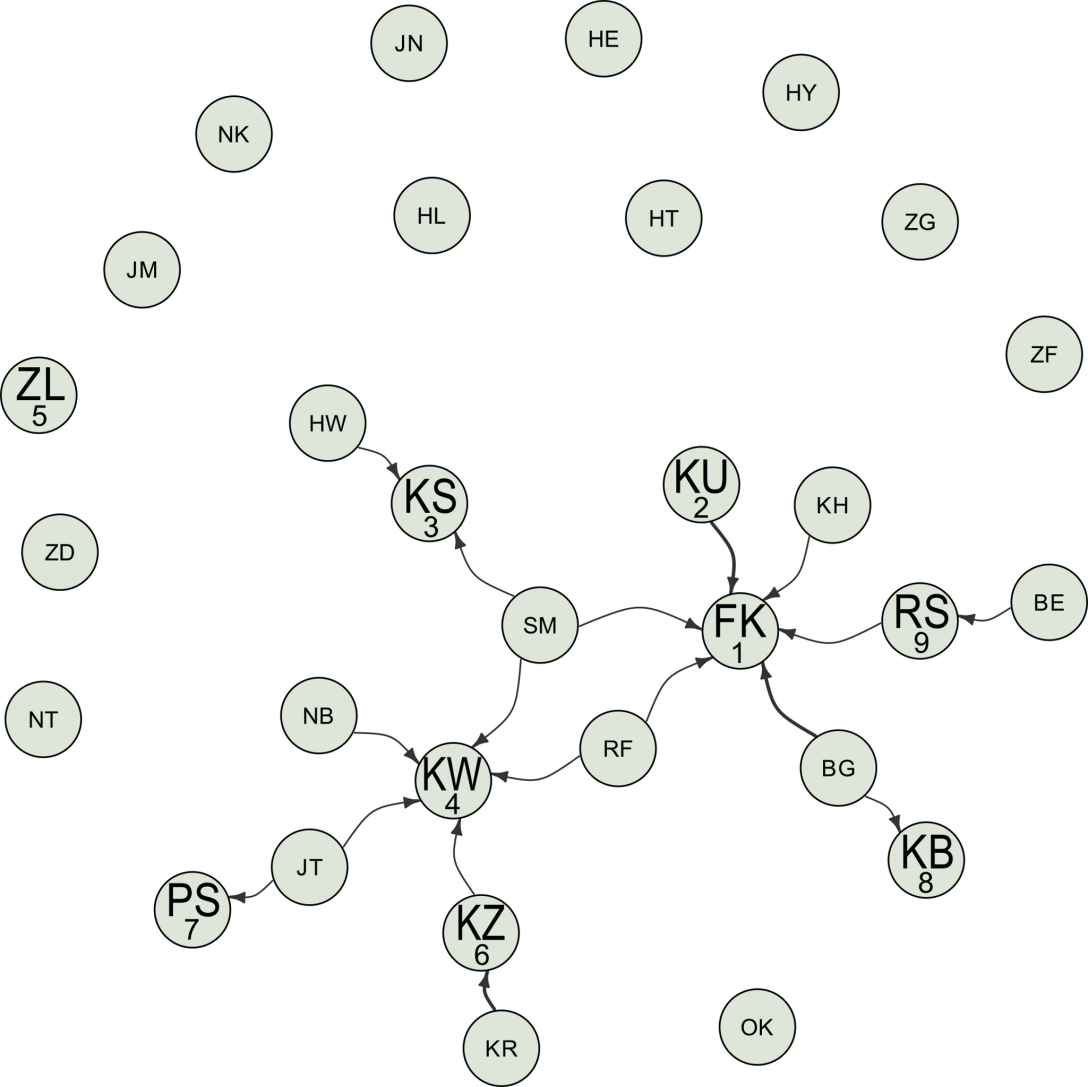
Visualization of the static interaction networks for the RU1 behavior for all 30 individuals. Graphs are laid out using the Fruchterman–Reingold weighted algorithm. Labels on the nodes indicate the identity of individuals (see Supporting Information). Individuals with large label size developed the behavior, whereas individuals with small label size did not. Numbers under the large label indicate the order of acquisition of the behavior. The width of the arrows linking individuals is proportional to the number of times an interaction event was recorded between any two individuals and represented according to the convention “X→Y” means that Y was observed by X. Data were deposited in the Dryad repository: http://dx.doi.org/10.5061/dryad.m6s21.

Leaf-sponging in chimpanzees is considered a behavioral universal [10], but there is considerable variation in how this technique is used in different communities [38]. The behavior is customary in the Sonso community, where most chimpanzees of all age classes display the behavior [10]. Sonso chimpanzees typically manufacture leaf-sponges (LSs) by folding and chewing leaves in their mouth, subsequently using them in water sources to drink [39] or, in experimental conditions, to collect honey [21]. In November 2011, members of the community visited a novel sponging site, a recently flooded waterhole located in swamp forest adjacent to a seasonal river. During 6 d of continuous observation, various individuals were observed to develop two tool behaviors, novel to the group: “leaf-sponge re-use” and “moss-sponging.” Both behaviors spread partially through the group (Figure 1 and 2). We defined “moss-sponging” (M) as the production of a sponge consisting entirely of moss or a mixture of leaves and moss. Moss-sponging, while rare, has been previously documented in one other chimpanzee community [40] and one bonobo community [13]. We defined “leaf-sponge re-use” (RU1) as utilizing a previously fabricated and used sponge that had been *discarded* on a previous visit, in contrast to standard leaf-sponging where an individual collects leaves from a branch. A second, more common type of leaf-sponge re-use (RU2) consisted of infants obtaining a sponge directly from an older relative by begging or scrounging (see Materials and Methods). RU1 has been previously reported in one other chimpanzee community but only in infants and juveniles [38].

The Sonso chimpanzees have been under continuous observation for the last 20 years, with regular observations of LS and RU2 but no recorded observations of RU1 or M, suggesting that we observed the initial spread or “diffusion” of two innovations to their tool repertoire [41].

The two novel behaviors emerged in an unusual ecological context, the discovery of a waterhole that had been repeatedly flooded by the river. By analyzing in parallel the spread of the two behaviors and comparing the two groups of individuals who learned them, we could determine whether the environment alone could explain the spread or whether there was an added effect of social learning. To this end, we monitored the exact party composition of all individuals present at the waterhole, which allowed us to identify who observed whom performing the novel behaviors and to construct the corresponding social network models. The different models were fitted to the data by maximum likelihood and tested against models with no social transmission, using corrected Akaike's Information Criterion for small sample size (AIC_c_). We included potentially confounding factors (age, gender) to investigate their effects on learning rates (see Materials and Methods) [7],[8]. We considered a number of functional forms (see Materials and Methods) for the relationship between the number of observations and the rate of learning within the models fitted to the order in which individuals learned each behavior (Order-of-Acquisition Diffusion Analysis, OADA) and models fitted to the times at which they learned (Time-of-Acquisition Diffusion Analysis, TADA). Here, we only present the results from the best dynamic network order of acquisition model (see Supporting Information for details of all models fitted). In the best model, the number of observations of the target behavior had a log-linear relationship with the rate at which that behavior was learned; that is, each observation increased it by a specific ratio. We used an information theoretic approach using AIC_c_ to compare the predictive power of dynamic and static networks and assess the evidence for social transmission.

## Results

Our analysis starts with the alpha male NK extracting water from the waterhole and fabricating a moss-sponge (M, November 14, 2011; 9:05 a.m.), while being observed by the adult dominant female NB. Over the following 6 d period, the waterhole was revisited regularly and we observed a further seven individuals fabricating and using moss-sponges (M). For six of them, we could establish that they had observed M before (see “Audience” criterion in Materials and Methods). For the seventh individual, the dominant adult female KW, we could not confirm this, and we treat her as having independently innovated M (November 16, 2011; 9:07 a.m.), although this happened less than 1 min after having re-used another chimpanzee's discarded moss-sponge (Figure 1).

Also on the 14th, subadult male FK retrieved and used a discarded LS (RU1). A further eight individuals developed the RU1 behavior, but four of them did so apparently without having observed another individual performing this behavior (Figure 2).

Neither MS nor RU1 had previously been recorded in the Sonso community, and we employed NBDA to analyze the patterns of transmission over the 6-d period. The dynamic network NBDA had 12.3× more support than the static network NBDA. Therefore, we report estimates of the effect of social transmission from this model, although results were qualitatively similar for both models (see Supporting Information). For both dynamic and static networks, there was most support for models with social transmission of moss-sponging but not RU1 (dynamic, Total Akaike weight, Σw_i_ = 0.754; static, Total Akaike weight, Σw_i_ = 0.801), in particular when comparing the support for these models to the support of models with no social transmission of either behavior (dynamic, 600,000×; static, 18,000×; Table 1). The estimated social transmission effect for moss-sponging was an 14.9× increase in learning rate for each observation of an informed individual performing moss-sponging (95% C.I., 4.7 to 88.2; Table 2), corresponding to an estimated 84.5% acquiring moss-sponging by social transmission (excluding the innovator). However, this is conservative: One individual (KW) acquired moss-sponging without any evidence of first observing another individual; thus, NBDA assumes she could not have done so with social transmission. However, KW acquired M after re-using another chimpanzee's sponge that contained moss, suggesting social learning mediated through the products of the moss-sponging behavior, a pathway the network was not intended to capture. With KW's acquisition excluded, the effect of an observation is estimated to be a 21.2× increase in learning rate (95% C.I., 4.2 to 679), corresponding to an estimated approximate 99.1% acquiring M by social transmission. An additional analysis suggests that it is highly unlikely that the social transmission effect for M is an artifact caused by differential exposure to the waterhole (see Supporting Information).

**Table 1:** Total Akaike weight (support) for different models of social transmission of moss-sponging (M) and LS re-use (RU1), assuming (a) a static network and (b) a dynamic network.

Social Transmission Model	Total Akaike Weight (Σw_i_)	
	(a) Static Network	(b) Dynamic Network
1. Asocial learning	1.38×10^−5^	1.12×10^−6^
2. Same social transmission effect	0.096	0.0002
3. Different social transmission effect	0.397	0.246
4. Social transmission of M only	0.603	0.754
5. Social transmission of RU1 only	1.27×10^−5^	6.37×10^−7^

**Table 2:** Estimates of (a) social transmission effects for LS re-use (RU1) and moss-sponging (M) variants, giving the multiplicative effect on learning rate of each observation (1×, no effect); (b) the ratio of social transmission effects between M and RU1; and (c) the estimated number of acquisitions that were by social transmission, excluding the innovation event.

	(a) Social Transmission (Multiplicative Effect Per Observation)	(b) Ratio: M Effect/RU1 Effect	(c) % of Events by Social Transmission
RU1	1.07×(0.58–2.48)	—	3% (0%*–19%)
Moss-sponging KW included	14.93×(4.67–88.24)	2.42×(4.67–72.24)	85% (80%–86%)
Moss-sponging KW excluded	21.17×(4.19–679)	15.90×(3.00–230)	99% (92%–100%)

Estimates are model-averaged estimates, with unconditional confidence intervals in parentheses. For M, estimates are given both with KW included (conservative estimate) and excluded (see text for explanation).

*Note that the lower 95% C.I. limit for the social effect on RU1 is <1, meaning each observation *decreases* the rate of learning; we set this situation to be zero events by social transmission.

Though we cannot rule out social transmission of RU1 (see Table 2), effects were weaker than for M (Σw_i_ = 0.246; compared with the same social effect, Σw_i_ = 0.0003; Table 1). The social effect on moss-sponging was conservatively (i.e., with KW included) estimated to be 11.3× stronger than the social effect on RU1 (95% C.I., 4.67 to 72.24). The estimated social transmission effect for RU1 was only an increase of 1.07× for each observation (95% C.I., 0.58 to 2.48), corresponding to an estimated approximate 3.1% acquiring RU1 by social transmission. Taken together, our results demonstrate a social transmission effect for M and a weak social transmission effect for RU1.

## Discussion

We have applied a novel form of network analysis to investigate the spread of two novel tool-use behaviors with the same function, which has produced evidence for social learning. The observed patterns of diffusion indicated that visiting a new resource jointly was not sufficient to explain the spread of M by individual learning, but that individuals influenced each other during acquisition. Our analyses also made it unlikely that some unknown variable influenced both the network structure and the rate at which individuals acquired M. In contrast, we found strong evidence for a social effect on the diffusion of M and a weak one for RU1, indicating that social learning plays a role in the transmission of novel behaviors in wild chimpanzees.

What factors could have favored the emergence of the two novel behaviors? In our case, moss-sponging was unlikely to have been invented because of a scarcity of leaves, which were widely available (see Supporting Information). Moreover, Sonso chimpanzees have regularly been observed manufacturing LS at other clay-pits, presumably to access minerals (Reynolds V, Lloyd AW, English CJ, Lyons P, Dodd H, et al., Budongo Forest chimpanzees' sodium resources: New adaptations, unpublished manuscript), but no moss-sponging has ever been documented, despite moss also being widely available. Similarly, although chimpanzees routinely abandon LSs in and around tree holes throughout the forest, RU1 has never been observed (although RU2 is common). A possible factor is that this site appeared to attract larger groups and foster greater competition than that which has usually been observed at water sources, potentially because of unusually high mineral levels (Reynolds V, Lloyd AW, English CJ, Lyons P, Dodd H, et al., Budongo Forest chimpanzees' sodium resources: New adaptations, unpublished manuscript). It is plausible that the high levels of competition at the new site favored innovation of moss-sponging. However, increased physical proximity alone could not explain the subsequent spread of the behavior in the group, as both moss and leaves were collected within 5 m from the waterhole, and leaf-sponging remained more frequent than moss-sponging (see Supporting Information), possibly due to chimpanzees' conservatism [42]–[45]. The emergence of RU1 may reflect increased opportunities for encountering other chimpanzees' leave-sponges. This interaction with discarded sponges could be interpreted as a kind of social learning, not influenced by direct observations, but akin to local or stimulus enhancement [46]. However, it is unclear what specific information could be retrieved: a discarded re-used sponge does not yield the information that it has been or may be “re-used” relative to a sponge that has only been used once. Only in the re-use of a moss-sponge by KW did some information appear to be gained: that moss can be employed as a sponging material, as she subsequently developed moss-sponging. In contrast, our NBDA analysis showed that the subsequent diffusion of moss-sponging occurred along the innovators' social network. Although ecological factors may have provided favorable conditions for the initial innovation of moss-sponging, this cannot explain why, in the absence of social transmission, eight chimpanzees converged on the solution within 6 d. Instead, it seems likely that while its innovation was ecologically driven, the spread through the group was a result of social transmission, paralleling findings in early hominins [47].

Our study adds new evidence supporting the hypothesis that some of the behavioral diversity seen in wild chimpanzees is the result of social transmission and can therefore be interpreted as cultural, especially when considered together with previous results from the wild [10] and captivity [48]. Our findings were made possible by employing a novel version of the NBDA that can incorporate information about the time course of the recorded observations. In doing so, our model captures a key aspect of social learning—that is, who observed the novel behavior at what time and from whom. In previous static versions of NBDA, for example in humpback whales, long-term association patterns were used to infer who had observed whom [8]. Our dynamic model requires fully habituated individuals that can be closely observed [49]; however, where this is possible, it is more powerful, as is demonstrated by the fact that dynamic networks were more supported than corresponding static networks.

Previous studies of vervet monkeys [50],[51] and captive chimpanzees [52] have found an influence of the model's rank on diffusion of behavior. Although our small sample size did not allow us to analyze rank effects, moss-sponging was first invented by the alpha male before spreading to two other individuals. And similarly, the second inventor, KW, was dominant over all the individuals who learned moss-sponging from her [53]. As all individuals appeared to develop the behavior directly after having observed it, it was not possible to make inferences on whether dominant individuals transmitted the behavior more effectively than others. Nevertheless, it is interesting to note that the social learning effect was less marked in RU1, which was first demonstrated by lower ranking individuals.

Although our results suggest social learning of moss-sponging via direct observation, the nature of the social learning mechanism remains unclear [54],[55]. Chimpanzees display a range of social learning mechanisms, including emulation and imitation [28]–[34], similarly to some monkey species [54],[55]. Teaching and imitation are often said to be central in the diffusion of human culture [35],[36],[56],[57], but other social learning mechanisms can also generate behavioral traditions [31],[58]. For example, early hominins who contributed to the Oldowan technology (2.6 mya) may have relied on emulative processes rather than imitation [47],[59]–[61], in contrast to the later Acheulean technology [62],[63]. However, as our results do not allow us to identify the precise learning mechanism employed during the social transmission of moss-sponging, it remains possible that this may vary from those on which humans rely to transmit their culture. Until the precise nature of these learning mechanisms is established, questions will remain about potential evolutionary discontinuity in the transmission of “cultural” behavior [36].

Nevertheless, although social learning mechanisms are important, our data support a growing literature that refutes a strong distinction between individual and social learning. Both rely on the same basic understanding of physical cognition and only differ in the presence or absence of a task-related social memory [64]. How existing techniques were modified and what was transmitted may have been equally important in the first stages of human evolution. In our view, further progress in the study of animal culture must go beyond the surface behavioral level, as is usually practiced, and address the cognitive and neural processes involved during innovation and social transmission [65]. For instance, both re-use and moss-sponging appear to be modifications of existing behaviors, rather than fully novel innovations. Observers may have been aided by an already existing mental representation when acquiring the novel behavior [66],[67] that they updated after observing knowledgeable individuals [68],[69]. Studying these processes in more detail in our closest relative and other animals may thus inform our understanding of early hominin culture and the evolutionary processes that eventually led to modern human cultures [70]–[72].

## Materials and Methods

### Ethics Statement

Permission to conduct this research was given by the Uganda Wildlife Authority (UWA), the Ugandan National Council for Science and Technology (UNCST), and the National Forestry Authority (NFA).

### Study Site and Subjects

The Budongo Conservation Field Station was established in 1990 in the Budongo Forest Reserve, which lies in the western Rift Valley in Uganda (1°350–1°550 N, 31°180–31°420 E) at a mean altitude of 1,050 m. The 793 km^2^ Reserve includes 482 km^2^ of continuous medium-altitude semideciduous forest cover. The Sonso community has been under continuous observation since the early 1990s with individuals individually known and habituated to human observers for about 20 y [39].

During data collection in November 2011, the Sonso study community of chimpanzees consisted of 68 named individuals. Following Reynolds [39], we defined age groups as infants (0–4 y), juveniles (5–9 y), subadults (m, 10–15 y; f, 10–14 y), and adults (m, 16+ y; f, 15+ y). Using these categories, the group composition was 30 adults (10 males and 20 females), 15 subadults (4 males and 11 females), 13 juveniles (4 males and 9 females), and 10 infants (3 males and 7 females).

### Procedure

#### Data Collection and Site Location

Data were collected on November 14–19, 2011, between 7 a.m. and 5 p.m., at a socially contested waterhole between the roots of two trees (*Cynometra alexandrii* and *Mimusops bagshawei*) located in an area of recently flooded swamp forest approximately 5 m from a seasonal river (Figure S1). The hole contained high mineral levels compared with other nearby water sources, such as the river (Na, K, Ca, Mn, Cl) (Reynolds V, Lloyd AW, English CJ, Lyons P, Dodd H, et al., Budongo Forest chimpanzees' sodium resources: New adaptations, unpublished manuscript). All observed cases of leaf-tool fabrication and use were recorded using a hand-held high-definition camcorder (Panasonic HD60) [73].

#### Sponge Material

Although leaf-sponging was focused on the waterhole, there were a number of additional stagnant puddles within a 3-m radius where individuals used LS tools and drank directly (Figure S2). Leaves used to manufacture sponges were identified as *Lasiodiscus mildbraedii*, *Lychnodiscus cerospermus*, and *Agromolera* subspecies. Mosses were collected in the waterhole area when chimpanzees were absent. Species were identified as *Pilotrichella cuspidate*, *Racopilum africanum* (*Mitt*), and *Pinnatella minuta* (*Mitt*). Additionally, two liverwort species, *Plagiochila strictifolia* (*Steph*) and *Plagiochila pinniflora* (*Steph*), were identified. These primitive plants looked similar to flattened mosses and may have been part of the moss-sponges.

#### Definitions

Following Whiten et al. [11], LS is “wad of leaves/vegetation chewed and used to collect water, then squeezed in mouth.”

Moss-sponge, following Lanjouw [40], is defined as follows: “chimpanzees collected moss off the bark of the trees, loosely rolled it into a bundle, generally not bigger than a few centimeters wide.” Moss-sponge was inserted into the mouth at least once before sponging. In both previous cases, the sponges appeared exclusively composed of moss despite leaves being freely available. In Sonso, moss may be combined, but not necessarily, with leaves in the initial fabrication or added to an existing LS (Videos S1 and S2).

Fabrication is the removal/collection of leaves or moss and fabrication of sponge in mouth, but sponge is not subsequently dipped into water, for example, as access to the sponging location is blocked by another individual.

Use is defined as dipping of sponge into water and insertion at least once into mouth to suck the water.

Re-use (type 1 and 2) is defined as follows: We coded as re-use type 1 (Video S3) the recovery of a used sponge that had been fabricated by another individual (or possibly by the same individual on a previous visit to the sponging location) and discarded. We distinguished this from re-use 2, a commonly observed behavior in which infants beg or scrounge for sponges made by their mother or older maternal siblings, as this is done while the older relative is using the sponge, as opposed to after they have discarded it (Video S4). In Sonso, RU2 appears limited to immature individuals and has never been recorded in mature individuals. Similarly, in West African chimpanzees (*P. t. verus*), both RU1 and RU2 are observed, but the behavior is only displayed by infants and juveniles [38].

Drinking is defined as drinking directly with the mouth from the water source.

#### Coding

Video files were uploaded to an Apple MacBook Pro using iMovie and edited into discrete clips for analysis. We coded the following variables for all occurrences of leaf-tool fabrication, (re-)use, and direct drinking: date, individual identity, party composition, specific audience (individuals within 1 m), fabrication of sponge (removal of material and fabrication of sponge in mouth, collection of discarded sponge from the ground), use of sponge for drinking (sponge dipped in water and back to mouth at least once), sponge material (leaf or moss), and location (sponging-hole or puddle).

#### Audience

Individuals within 1 m of the model while the model was fabricating the sponge, but excluding individuals with either their head turned fully away or with their view obstructed by the environment (for example, sitting behind a tree-buttress or with their head inside the waterhole), were considered to be “potential observers.”

A second more restrictive definition was also applied for the “specific audience” in which individuals had to be within 1 m of the model and were considered to have actively looked at the model while the sponge was fabricated. This specific audience included individuals who were seen to shift their eye gaze to the model or to track the model's movements with their head movements or who had their head facing the model ±45° (as per [74]).

#### Network Reconstruction

A separate network was constructed for M and RU1. In each case, a directed edge was considered to exist between two individuals, from X to Y if there was at least one registered occurrence of X observing Y performing the RU1 or M behavior prior to X acquiring the relevant behavior themselves. The latter criterion was included as behavior can only be transmitted by observations that occur prior to acquisition of behavior and such that a positive result could not be indicative of homophily—that is, individuals who acquire a behavior being subsequently attracted to one another and thus observing each other more. The weight of the directed edge, a_YX_, was equal to the number of such occurrences.

For the dynamic social network, the edges were allowed to vary over time. Here, a_YX_(t) was taken to be the number of times X had observed Y performing the target behavior prior to time t. We also considered a binary dynamic network, where a_YX_(t) was taken to be 1 if X had observed Y performing the target behavior prior to time t, and 0 otherwise. We included this to allow for the possibility that a single observation of the target behavior may be sufficient for a maximal social transmission effect to occur.

### Statistical Analysis

To analyze the spread of the behaviors, we entered information about all individuals who used at least one tool at the tree-hole in NBDA models (*N* = 30). We ran an OADA [2] treating M and RU1 as independent diffusions included in the same model, allowing us to test for difference in the social transmission effect. We used the R script model for NBDA Version 1.2.11 available at http://lalandlab.st-andrews.ac.uk/freeware.html.

NBDA is based on survival analysis models and so assumes that the spread of the behavior is a stochastic process and that a naïve individual, *i*, has at any time a given learning rate, 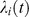, for each behavior pattern in question. We included a number of potentially confounding variables: x_1_, age (in years); x_2_, time spent in the community (in years); x_3_, sex (0/1 for female/male, respectively). These data were extracted from the Sonso community official list of individuals downloaded at http://www.budongo.org/. There is little support for an important effect of any individual-level variable (see Table S2). We considered both conventional NBDA models with the static social network and expanded the approach to include the dynamic network described above. For the static network NBDA, there are two functional forms for inclusion of individual-level variables in an NBDA [2], a model in which the interaction between social transmission and the individual-level variables is taken to be additive:
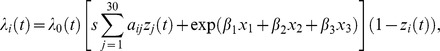
and one in which it is taken to be multiplicative:
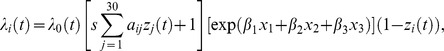
where 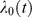 is a baseline rate function, which in OADA remains unspecified; s is the effect of social transmission per occasion *i* observed *j*; 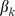 is the multiplicative effect of individual-level variable *k* on the log scale; and *z_i_(t)* is an indicator variable that takes the value 1 if *i* has acquired the behavior by time *t* and 0 otherwise. Both additive and multiplicative models were fitted: Findings were similar for each, but the multiplicative model had slightly better support (see Table S1), as reported in the main text.

The log-likelihood for acquisition event *l*, occurring at time *t_l_*, at which individual *m* acquired the behavior is:
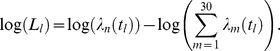



The log-likelihood for the whole diffusion is calculated by summing across all acquisition events. In a reanalysis, we excluded the M acquisition event for KW (see main text) by simply excluding this acquisition event from the likelihood function.

Proportion of acquisitions that were by social transmission was estimated for the best model (with no individual-level variables) by calculating for each acquisition event *l*>1:
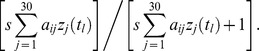



Here, the numerator is the rate of social transmission relative to the rate of asocial learning at time of the *l*-th acquisition event, and the denominator is the total rate of learning relative to the rate of asocial learning. Therefore, the whole equation gives the probability that event *l* occurred by social transmission, predicted by the model. By averaging across all acquisition events except the initial acquisition, we obtain the estimated proportion of events (excluding the innovation) that occurred by social transmission.

A static network based on observations does not fully allow for the time course of observations. To illustrate, one can imagine a group of three individuals: A, B, and C. A learns the behavior first. Next, B observes A performing the behavior three times and then learns the behavior. Finally, C observes A performing the behavior three times and subsequently learns the behavior last. A static network would represent the network as having links of strength 3 from A to both B and C, so an NBDA model based on such a network would predict that B and C were equally likely to learn second. In fact, we would expect B to be more likely to learn second, because B observed A performing the behavior first. A dynamic network allows us to incorporate this information into the NBDA.

We considered a number of different functional forms for the dynamic network. First, we considered a model in which each successive observation of the target behavior had a linear relationship with the rate of learning. As with the static network NBDA, we considered models in which the interaction with individual-level variables was taken either to be additive or to be multiplicative. These models are identical to those given above, except a_ij_ is replaced with a_ij_(t). We also considered a form where the effect of each successive observation of the target behavior had a linear effect on the log scale, on the rate of learning—that is, each successive observation multiplied the rate of learning by exp(s):
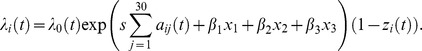



We refer to this as the log-linear model. Here a single observation adds *s* to the linear predictor [inside the exp() term] having the effect of multiplying the rate of learning by a factor of *exp(s)*. We also considered a version of the log-linear model in which the interaction with individual-level variables was additive:
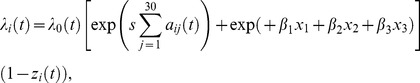
but this had less support than the multiplicative version (see Table S1).

For our dynamic network, the log-linear model is equivalent to including the number of observations of the target behavior prior to time t as a time-varying covariate in a Cox model [75]. This allowed us to use the survival package [76] to fit the models in the R statistical environment [77] to include a random (or frailty) effect to account for the fact that each diffusion included the same individuals. However, the random effect was estimated to be negligible and had no effect on the results, corresponding to the fact that each behavior diffused through a different subset of the group (with the exception of KW). Consequently, we dropped the random effect from the analysis. The model using the binary dynamic network is specified using the same equation as the log-linear model. The likelihood function given above for the static network NBDA is valid for all models given here.

Analogously to the linear model, the proportion of acquisitions that were by social transmission was estimated for the best log-linear model (with time in population included) using the dynamic network by calculating for each acquisition event *l*>1:
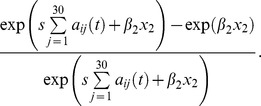



Here the numerator is the estimated rate of learning at the time of acquisition of the behavior minus the rate that would be expected under asocial conditions, and so can be thought of as the rate of social transmission. The denominator is the total rate of learning at the time of acquisition, so the fraction gives the probability the event occurred by social transmission. Averaging across all acquisition events except the initial innovation gives the estimated proportion of acquisitions that were by social transmission, excluding the innovation, which is known not to have occurred by social transmission.

We used an information theoretic approach using Akaike's Information Criterion corrected for sample size (AIC_c_) to allow for model selection uncertainty. This allowed us to estimate the support for each variable/model of social transmission, calculate model-averaged estimates of effects, and construct unconditional confidence intervals using profile likelihood methods [78].

### Time of Acquisition Diffusion Analysis

Because the TADA can have more statistical power than OADA [2], we fitted TADA models to check the robustness of our findings. The times of learning entered into the models were the cumulative time across days, including only times at which the group was present at the waterhole—to allow for the fact that the rate of learning would be zero when the group was not present at the waterhole. We fitted models assuming a constant baseline function 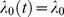, and models allowing for the possibility that 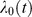 might systematically increase or decrease over time [79]. We also fitted models in which the baseline rate differed between M and RU1, to allow for differences in the asocial rate of learning. For the TADA analysis, the best model was the standard linear form of NBDA: Here we report the results of this set of models, though other functional forms gave similar results. For many models, the estimated Hessian matrix could not be inverted, so we could not reliably extract standard errors, meaning we could not calculate confidence intervals allowing for model selection uncertainty [78]. Consequently traditional confidence intervals are reported for TADA—that is, conditional on the best model containing the relevant parameter.

There was stronger evidence for social transmission of RU1 (same social effect as for M, Σw_i_ = 0.289; different social effects, Σw_i_ = 0.268) though still more support for social transmission of moss-sponging only (Σw_i_ = 0.443). For moss-sponging, s was estimated at 42.5 (95% C.I. = 6.74–814). corresponding to 84.3% (77.5%–85.6%) of acquisition events by social transmission, excluding the innovator. For leaf-sponging re-use, s was estimated to be 1.18 (95% C.I. = 0–6.78) corresponding to 22.3% (0%–36.4%) of acquisition events occurring by social transmission. The difference in s parameters (M – RU1) was estimated to be 41.3 (95% C.I. = 5.16–800). Therefore, the results of the TADA are qualitatively similar to the results of the OADA. In the main text, we present the results of the OADA as it makes fewer assumptions about the underlying baseline rate: although we can allow for a systematically increasing baseline rate using TADA, it is difficult to allow for a fluctuating rate, caused by changing conditions in the environment—for example, temperature changes affecting motivation to drink [33]. Consequently, we suspect OADA is likely to be more reliable in uncontrolled conditions.

### Strict Observation Criterion

To assess the robustness of our findings to the judgments we made about who observed whom, we repeated both OADA and TADA analyses using static and dynamic networks based on a stricter criterion of recording observation (see above). Overall the strict network had 0.43× less support than the less strict network for OADA, and slightly more support for TADA (1.2×). In both cases, the Akaike weights showed a similar pattern of support using each observation criterion (see Table S1 and Figures S4 and S5).

Note that both (a) recording of nonobserving individuals as observers and (b) failure to record observers will obscure any existing relationship between the observation network and the pattern of diffusion. This has two consequences: First, a stricter observation criterion does not necessarily mean a more accurate estimate of s parameters, as it may reduce cases of a but at the potential cost of increasing cases of b. Second, in either case, the effect of such errors in recording will be a tendency to *underestimate* social transmission effects, so the reported social transmission of M could not be the result of a bias arising from errors in recording who observed whom.

### Controlling for Exposure to the Waterhole

A potentially confounding variable is the different level of exposure each chimpanzee had to the waterhole. *A priori*, it seemed possible that chimpanzees that interacted with the waterhole more frequently would be more likely to acquire both behavior patterns than chimpanzees that interacted with the water hole less frequently. If this exposure was correlated with observation of others performing M, this could create a spurious social transmission effect. To an extent, the different level of social transmission for M and RU1 weakens the case for this explanation, as we would expect an exposure effect to operate similarly on both behavior patterns. Nonetheless, we ran additional analyses to allow for the potential effects.

We calculated an exposure score for each chimpanzee for each behavior pattern as being the rate at which each chimpanzee interacted with the waterhole—that is, initiated bouts of normal leaf-sponging behavior. If a chimpanzee did not acquire the behavior pattern in question (M or RU1), exposure was calculated over the whole period for which we observed the chimpanzees at the waterhole ( = number of interactions/total observation time). For chimpanzees that acquired a behavior pattern, the corresponding exposure score was calculated over the time preceding acquisition of that behavior (e.g. = number of interactions prior to acquiring M/time at which M was acquired), as exposures experienced after acquiring M (for example) cannot exert a causal effect on the acquisition of M.

We first added exposure score as a predictor to the best model for the OADA reported in the main text, with exposure constrained to have the same effect on both M and RU1. This model had 0.43× less support, the effect of exposure was estimated to be small, and the estimate of the social transmission parameter remained very similar (s = 2.79). We then wished to allow for the possibility that exposure might affect only M, thus resulting in a spurious social transmission effect for M. This model had 3.92× more support than the previous best OADA model. However, contrary to expectations, the effect of exposure was estimated to be negative with a 9.3% reduction in rate of acquisition for one standard deviation difference in exposure score (see Figure S6). Most importantly, the effect of social transmission was estimated to be slightly higher in this model (s = 3.00), suggesting that differential exposure to the waterhole is unlikely to have resulted in a spurious social transmission effect for M.

## Supporting Information

Figure S1 Location of the waterhole between the roots of the two trees (photo by Nina Hänninen, with permission).
(TIF)

Figure S2 Broad view of the two trees (right, individual NB) and the puddles (left, individual OK) at the sponging location (photo by Catherine Hobaiter).
(TIF)

Figure S3 Proportion of individuals using different techniques at the waterhole (November 14–19). Drink, drink directly from the hole; Alternative, proportions of moss and re-use 1 combined.
(PDF)

Figure S4 Visualization of the interaction networks for the moss-sponging behavior for all 30 individuals, in the case of the specific audience, using a stricter observation criterion (see Materials and Methods). Graphs are laid out using the Fruchterman–Reingold weighted algorithm. Labels on the nodes indicate the identity of individuals. Individuals with large label size developed the behavior, whereas individuals with small label size did not. Numbers under the large label indicate the order of acquisition of the behavior. The width of the arrows linking individuals is proportional to the number of times an interaction event was recorded between any two individuals and represented according to the convention “X→Y” means that Y was observed by X. Dashed line, potential product-based social learning by individual KW who re-used a moss-sponge. Data were deposited in the Dryad repository: http://dx.doi.org/10.5061/dryad.m6s21.
(TIF)

Figure S5 Visualization of the interaction networks for the RU1 behavior for all 30 individuals, in the case of the specific audience, using a stricter observation criterion (see Materials and Methods). Graphs are laid out using the Fruchterman–Reingold weighted algorithm. Labels on the nodes indicate the identity of individuals. Individuals with large label size developed the behavior, whereas individuals with small label size did not. Numbers under the large label indicate the order of acquisition of the behavior. The width of the arrows linking individuals is proportional to the number of times an interaction event was recorded between any two individuals and represented according to the convention “X→Y” means that Y was observed by X. Data were deposited in the Dryad repository: http://dx.doi.org/10.5061/dryad.m6s21.
(TIF)

Figure S6 Boxplot showing the rate of interaction with the waterhole—that is, initiation of bouts of normal leaf-sponging (exposure) for chimpanzees that did and did not acquire moss-sponging behavior. For those that did, the rate of interaction is calculated prior to their acquisition of moss-sponging.
(EPS)

Figure S7 Location of the sponging site on the Budongo Conservation Field Station Grid System.
(TIF)

Table S1 Akaike weights for different social learning models assuming static or dynamic networks; with a linear, binary, or log-linear relationship with the rate of learning; with an additive or multiplicative interaction with individual level variables; and in which there was (a) equal social transmission for M and RU1, (b) differing levels of social transmission for M and RU1, (c) social transmission for M only, and (d) social transmission for RU1 only, compared with the Akaike weight for an asocial model. Akaike weights do not sum to 1 because a model with no individual-level variables qualifies as both an additive and multiplicative model. However, each cell represents the same number of models so the weights are directly comparable between cells. The upper panel corresponds to the analysis presented in the main text: Here the two cells with highest support account for 75% of the total support between them. Akaike weights are similar when KW's M acquisition event is excluded. The lower panel corresponds to the analysis based on the strict observation criteria and shows a similar pattern of results.
(DOC)

Table S2 Summary of results for individual-level variables, from the log-linear model using the dynamic network. Effects are given on the log scale with Wald confidence intervals calculated using the unconditional standard error.
(DOC)

Table S3 Pearson's correlation between techniques used by the chimpanzees and with days spent at the waterhole. LS, leaf-sponge; M, moss; RU1, re-use 1; ALT, alternative technique (M and RU1 combined); D, drink; DAY, days passed. To investigate whether the use of alternative techniques (M, RU1) was correlated to a decrease in available LS material, we ran Pearson's correlations using frequency of individual users per day per technique, and of each technique versus days passed, including drinking. If increased direct drinking were correlated with decreased users of LS, this may indicate an environmental constraint on tool production. There was no evidence of a correlation between the number of chimpanzees exhibiting the new techniques and the number of days passed (see Figure S3), suggesting that material availability did not influence tool choice. Furthermore, we found no correlations across days between the number of cases of LS and cases of either RU1, M, or RU1 and M combined, showing that selection of the techniques, old and new, were not associated. * In order to control for the varying number of individuals at the site per day, these tests are of number of individuals using the technique/total number of individuals, correlated against the number of days past. Degrees of freedom = 4 in all cases. All *p* values are two-tailed.
(DOC)

Table S4 List of Sonso individuals who manufactured at least one leaf-based tool at the waterhole in the course of the 6 d with individual information as of November 2011. Individual identity code, age (expressed in years), sex (F, female; M, male), age class, family (code of the mother), and tenure (time spent within the community expressed in years) are provided. Note that age and tenure estimates for individuals over 20 y are estimates and should be treated as ±3 y.
(DOC)

Movie S1 Innovation of the Moss-sponging behavior. NK gathers some moss on the tree trunk, while being observed by NB. He will then proceed to add some leaves to his sponge before leaf-sponging (video by Catherine Hobaiter).
(WMV)

Movie S2 Diffusion of the Moss-sponging behavior. NB gathers moss and adds it to her existing LS, before resuming leaf-sponging; she is observed by individual HL, who will display the behavior when she gets access to the waterhole (video by Catherine Hobaiter).
(MOV)

Movie S3 RU1 behavior. Individual KZ (right of the screen) picks an LS from the ground while his mother KW is extracting water from the waterhole. He then chews the used LS before leaf-sponging himself at the waterhole (video by Catherine Hobaiter).
(MOV)

Movie S4 RU2 behavior. Individual KS extracts an LS from his mother's mouth before using it at the waterhole (video by Catherine Hobaiter).
(MOV)

## Abbreviations

LS: leaf-sponge
M: moss
NBDA: network-based diffusion analysis
OADA: order-of-acquisition diffusion analysis
RU1: re-use type 1
RU2: re-use type 2
TADA: time-of-acquisition diffusion analysis

## References

[1] Franz M, Nunn CL (2009) Network-based diffusion analysis: A new method for detecting social learning. Proc Biol Sci 276: 1829–1836. 19324789 10.1098/rspb.2008.1824 PMC2674490

[2] Hoppitt W, Boogert NJ, Laland KN (2010) Detecting social transmission in networks. J Theor Biol 263: 544–555. 20064530 10.1016/j.jtbi.2010.01.004

[3] Dufour V, Sueur C, Whiten A, Buchanan-Smith HM (2011) The impact of moving to a novel environment on social networks, activity and wellbeing in two new world primates. Am J Primatol 73: 802–811. 21381071 10.1002/ajp.20943

[4] Pinter-Wollman N, Hobson EA, Smith JE, Edelman AJ, Shizuka D, et al (2013) The dynamics of animal social networks: analytical, conceptual, and theoretical advances. Behav Ecol 25 (2) 242–255.

[5] Wey T, Blumstein DT, Shen W, Jordán F (2008) Social network analysis of animal behaviour: a promising tool for the study of sociality. Anim Behav 75: 333–344.

[6] Kendal RL, Custance D, Kendal JR, Vale G, Stoinski T, et al (2010) Evidence for social learning in wild lemurs (*Lemur catta*). Learn Behav 38: 220–234. 20628161 10.3758/LB.38.3.220

[7] Claidière N, Messer EJE, Hoppitt W, Whiten A (2013) Diffusion dynamics of socially learned foraging techniques in squirrel monkeys. Curr Biol 23: 1251–1255. 23810529 10.1016/j.cub.2013.05.036

[8] Allen J, Weinrich M, Hoppitt W, Rendell L (2013) Network-based diffusion analysis reveals cultural transmission of lobtail feeding in humpback whales. Science 340: 485–488. 23620054 10.1126/science.1231976

[9] McGrew WC (1992) Chimpanzee material culture: Implication for human evolution. Cambridge, UK: Cambridge University Press.

[10] Whiten A, Goodall J, McGrew WC, Nishida T, Reynolds V, et al (1999) Cultures in chimpanzees. Nature 399: 682–685. 10385119 10.1038/21415

[11] Whiten A, Goodall J, McGrew WC, Nishida T, Reynolds V, et al (2001) Charting cultural variation in chimpanzees. Behaviour 138: 1481–1516.

[12] van Schaik CP, Ancrenaz M, Borgen G, Galdikas B, Knott CD, et al (2003) Orangutan cultures and the evolution of material culture. Science 299: 102–105. 12511649 10.1126/science.1078004

[13] Hohmann G, Fruth B (2003) Culture in Bonobos? Between-species and within-species variation in behavior. Curr Anthropol 44: 563–571.

[14] Rendell L, Whitehead H (2001) Culture in whales and dolphins. Behav Brain Sci 24: 309–324. 11530544 10.1017/s0140525x0100396x

[15] Madden JR (2008) Do bowerbirds exhibit cultures? Anim Cogn 11: 1–12. 17551758 10.1007/s10071-007-0092-5

[16] Laland KN, Janik VM (2006) The animal cultures debate. Trends Ecol Evol 21: 542–547. 16806574 10.1016/j.tree.2006.06.005

[17] Luncz LV, Boesch C (2014) Tradition over trend: Neighboring chimpanzee communities maintain differences in cultural behavior despite frequent immigration of adult females. Am J Primatol 76 (7) 649–657. 24482055 10.1002/ajp.22259

[18] Biro D, Inoue-Nakamura N, Tonooka R, Yamakoshi G, Sousa C, et al (2003) Cultural innovation and transmission of tool use in wild chimpanzees: Evidence from field experiments. Anim Cogn 6: 213–223. 12898285 10.1007/s10071-003-0183-x

[19] Lonsdorf EV (2006) What is the role of mothers in the acquisition of termite-fishing behaviors in wild chimpanzees (*Pan troglodytes schweinfurthii*)? Anim Cogn 9: 36–46. 16195914 10.1007/s10071-005-0002-7

[20] O'Mailey RC, Wallauer W, Murray CM, Goodall J (2012) The appearance and spread of ant fishing among the Kasekela chimpanzees of Gombe: A possible case of intercommunity cultural transmission. Curr Anthropol 53: 650–663. 25242820 10.1086/666943 PMC4166518

[21] Gruber T, Muller MN, Strimling P, Wrangham RW, Zuberbühler K (2009) Wild chimpanzees rely on cultural knowledge to solve an experimental honey acquisition task. Curr Biol 19: 1806–1810. 19853447 10.1016/j.cub.2009.08.060

[22] Luncz LV, Mundry R, Boesch C (2012) Evidence for cultural differences between neighboring chimpanzee communities. Curr Biol 22: 922–926. 22578420 10.1016/j.cub.2012.03.031

[23] Möbius Y, Boesch C, Koops K, Matsuzawa T, Humle T (2008) Cultural differences in army ant predation by West African chimpanzees? A comparative study of microecological variables. Anim Behav 76: 37–45.

[24] Humle T, Matsuzawa T (2002) Ant-dipping among the chimpanzees of Bossou, Guinea, and some comparisons with other sites. Am J Primatol 58: 133–148. 12454957 10.1002/ajp.10055

[25] Schöning C, Humle T, Möbius Y, McGrew WC (2008) The nature of culture: Technological variation in chimpanzee predation on army ants revisited. J Hum Evol 55: 48–59. 18275983 10.1016/j.jhevol.2007.12.002

[26] Koops K, McGrew WC, Matsuzawa T (2013) Ecology of culture: Do environmental factors influence foraging tool use in wild chimpanzees (*Pan troglodytes verus*)? Anim Behav 85: 175–185.

[27] Gruber T, Potts K, Krupenye C, Byrne M-R, Mackworth-Young C, et al (2012) The influence of ecology on chimpanzee cultural behaviour: A case study of five Ugandan chimpanzee communities. J Comp Psychol 126: 446–457. 22746159 10.1037/a0028702

[28] Whiten A, Spiteri A, Horner V, Bonnie KE, Lambeth SP, et al (2007) Transmission of multiple traditions within and between chimpanzee groups. Curr Biol 17: 1038–1043. 17555968 10.1016/j.cub.2007.05.031

[29] Hopper LM, Spiteri A, Lambeth SP, Schapiro SJ, Horner V, et al (2007) Experimental studies of traditions and underlying transmission processes in chimpanzees. Anim Behav 73: 1021–1032.

[30] Yamamoto S, Humle T, Tanaka M (2013) Basis for cumulative cultural evolution in chimpanzees: Social learning of a more efficient tool-use technique. PLoS ONE 8: e55768. 23383278 10.1371/journal.pone.0055768 PMC3559591

[31] Whiten A, McGuigan N, Marshall-Pescini S, Hopper LM (2009) Emulation, imitation, over-imitation and the scope of culture for child and chimpanzee. Philos Trans R Soc B Biol Sci 364: 2417–2428. 10.1098/rstb.2009.0069 PMC2865074 19620112

[32] Whiten A, Horner V, de Waal FBM (2005) Conformity to cultural norms of tool use in chimpanzees. Nature 437: 737–740. 16113685 10.1038/nature04047

[33] Bonnie KE, Horner V, Whiten A, de Waal FBM (2007) Spread of arbitrary conventions among chimpanzees: a controlled experiment. Proc Biol Sci 274: 367–372. 17164200 10.1098/rspb.2006.3733 PMC1702386

[34] Horner V, Whiten A, Flynn E, de Waal FBM (2006) Faithful replication of foraging techniques along cultural transmission chains by chimpanzees and children. Proc Natl Acad Sci U S A 103: 13878–13883. 16938863 10.1073/pnas.0606015103 PMC1564214

[35] Galef BG (2009) Culture in animals? In: Laland KN, Galef BG, editors. The question of animal culture. Cambridge, MA: Harvard University Press. pp. 222–246.

[36] Tomasello M (2009) The question of chimpanzee culture, plus postscript (Chimpanzee culture, 2009). In: Laland KN, Galef BG, editors. The question of animal culture. Cambridge, MA: Harvard University Press. pp. 198–221.

[37] Sayers K, Lovejoy CO (2008) The chimpanzee has no clothes: a critical examination of Pan troglodytes in models of human evolution. Curr Anthropol 49: 87–114.

[38] Sousa C, Biro D, Matsuzawa T (2009) Leaf-tool use for drinking water by wild chimpanzees (*Pan troglodytes*): acquisition patterns and handedness. Anim Cogn 12: S115–S125. 19697068 10.1007/s10071-009-0278-0

[39] Reynolds V (2005) The chimpanzees of the Budongo forest: Ecology, behaviour and conservation. Oxford, UK: Oxford University Press.

[40] Lanjouw A (2002) Behavioral adaptations to water scarcity in Tongo chimpanzees. In: C. Boesch, G. Hohmann, Marchant LF, editors. Behavioural diversity in chimpanzees and bonobos. Cambridge, UK: Cambridge University Press. pp. 52–60.

[41] Reader SM, Laland KN (2001) Primate innovation: Sex, age and social rank differences. Int J Primatol 22: 787–805.

[42] Gruber T, Muller MN, Reynolds V, Wrangham RW, Zuberbühler K (2011) Community-specific evaluation of tool affordances in wild chimpanzees. Scientific Reports 1: 128. 22355645 10.1038/srep00128 PMC3216609

[43] Hrubesch C, Preuschoft S, van Schaik CP (2009) Skill mastery inhibits adoption of observed alternative solutions among chimpanzees (*Pan troglodytes*). Anim Cogn 12: 209–216. 18766391 10.1007/s10071-008-0183-y

[44] Marshall-Pescini S, Whiten A (2008) Chimpanzees (*Pan troglodytes*) and the question of cumulative culture: An experimental approach. Anim Cogn 11: 449–456. 18204869 10.1007/s10071-007-0135-y

[45] Hopper LM, Schapiro SJ, Lambeth SP, Brosnan SF (2011) Chimpanzees' socially maintained food preferences indicate both conservatism and conformity. Anim Behav 81: 1195–1202. 27011390 10.1016/j.anbehav.2011.03.002 PMC4801479

[46] Thorpe WH (1956) Learning and instinct in animals. London: Methuen.

[47] Caruana MV, d'Errico F, Backwell LR (2013) Early hominin social learning strategies underlying the use and production of bone and stone tools. In: C. M. Sanz, J. Call, Boesch C, editors. Tool use in animals: Cognition and ecology. Cambridge, UK: Cambridge University Press. pp. 242–285.

[48] Whiten A, Mesoudi A (2008) Establishing an experimental science of culture: Animal social diffusion experiments. Philos Trans R Soc B Biol Sci 363: 3477–3488. 10.1098/rstb.2008.0134 PMC2607342 18799418

[49] McGrew WC (2004) The cultured chimpanzee: Reflections on cultural primatology. Cambridge, UK: Cambridge University Press.

[50] van de Waal E, Borgeaud C, Whiten A (2013) Potent social learning and conformity shape a wild primate's foraging decisions. Science 340: 483–485. 23620053 10.1126/science.1232769

[51] van de Waal E, Renevey N, Favre CM, Bshary R (2010) Selective attention to philopatric models causes directed social learning in wild vervet monkeys. Proc Biol Sci 277: 2105–2111. 20236972 10.1098/rspb.2009.2260 PMC2880145

[52] Horner V, Proctor D, Bonnie KE, Whiten A, de Waal FBM (2010) Prestige affects cultural learning in chimpanzees. PLoS ONE 5: e10625. 20502702 10.1371/journal.pone.0010625 PMC2873264

[53] Gruber T, Zuberbühler K (2013) Vocal recruitment for joint travel in wild chimpanzees. PLoS ONE 8: e76073. 24086688 10.1371/journal.pone.0076073 PMC3783376

[54] van de Waal E, Whiten A (2012) Spontaneous emergence, imitation and spread of alternative foraging techniques among groups of vervet monkeys. PLoS ONE 7: e47008. 23071698 10.1371/journal.pone.0047008 PMC3468485

[55] Voelkl B, Huber L (2007) Imitation as faithful copying of a novel technique in marmoset monkeys. PLoS ONE 2: e611. 17622356 10.1371/journal.pone.0000611 PMC1905941

[56] Richerson PJ, Boyd R (2005) Not by genes alone: How culture transformed human evolution. Chicago and London: University of Chicago Press.

[57] Hill K (2009) Animal “culture”? In: Laland KN, Galef BG, editors. The question of animal culture. Cambridge, MA: Harvard University Press. pp. 269–287.

[58] Caldwell CA, Schillinger K, Evans C, Hopper L (2012) End state copying by humans (*Homo sapiens*): Implications for a comparative perspective on cumulative culture. J Comp Psychol 126: 161–169. 22468937 10.1037/a0026828

[59] McPherron SP (2013) Perspectives on stone tools and cognition in the early Paleolithic record. In: C. M.Sanz, J.Call, Boesch C, editors. Tool use in animals: Cognition and ecology. Cambridge, UK: Cambridge University Press. pp. 286–309.

[60] Roche H, Blumenschine RJ, Shea JJ (2009) Origins and adaptations of early *Homo*: What archeology tells us. In: F. E.Grine, J. G.Fleagle, Leakey RE, editors. The first humans—Origin and early evolution of the genus *Homo*. Springer Netherlands. pp. 135–147.

[61] Backwell LR, d'Errico F (2008) Early hominid bone tools from Drimolen, South Africa. J Arch Sci 35: 2880–2894.

[62] Petraglia MD, Shipton C, Paddayya K (2005) Life and mind in the Acheulean. In: G.Gamble, Porr M, editors. The hominid individual in context. London & New York: Routledge. pp. 197–219.

[63] Porr M (2005) The making of the biface and the making of the individual. In: G.Gamble, Porr M, editors. The hominid individual in context. London & New York: Routledge. pp. 68–80.

[64] Heyes C (2012) What's social about social learning? J Comp Psychol 126: 193–202. 21895355 10.1037/a0025180

[65] Chittka L, Rossiter SJ, Skorupski P, Fernando C (2012) What is comparable in comparative cognition? Phil Trans R Soc B 367: 2677–2685. 22927566 10.1098/rstb.2012.0215 PMC3427551

[66] Gruber T, Singleton I, van Schaik CP (2012) Sumatran orangutans differ in their cultural knowledge but not in their cognitive abilities. Curr Biol 22: 2231–2235. 23142043 10.1016/j.cub.2012.09.041

[67] Bryson JJ (2009) Representations underlying social learning and cultural evolution. Interaction Studies 10: 77–100.

[68] Matsuzawa T, Biro D, Humle T, Inoue-Nakamura N, Tonooka R, et al. (2001) Emergence of culture in wild chimpanzees: Education by Master-Apprenticeship. In: Matsuzawa T, editor. Primate origin of human behavior and cognition. Tokyo, Japan: Springer. pp. 557–574.

[69] de Waal FBM (2001) The ape and the sushi master. London: Penguin.

[70] Whiten A, McGrew WC, Aiello LC, Boesch C, Boyd R, et al (2010) Studying extant species to model our past. Science 327: 410. 20093456 10.1126/science.327.5964.410-a

[71] McGrew WC (2010) In search of the last common ancestor: New findings on wild chimpanzees. Phil Trans R Soc B 365: 3267–3276. 20855301 10.1098/rstb.2010.0067 PMC2981959

[72] Toth N, Schick K (2009) The Oldowan: the tool making of early hominins chimpanzees compared. Annu Rev Anthropol 38: 289–305.

[73] Hobaiter C, Poisot T, Zuberbühler K, Hoppitt W, Gruber T (2014) Data from: Social network analysis shows direct evidence for social transmission of tool use in wild chimpanzees. Dryad Digital Repository Openly available via http://dx.doi.org/10.5061/dryad.m6s21. 10.1371/journal.pbio.1001960 PMC4181963 25268798

[74] Genty E, Breuer T, Hobaiter C, Byrne RW (2009) Gestural communication of the gorilla (*Gorilla gorilla*): repertoire, intentionality and possible origins. Anim Cogn 12: 527–546. 19184669 10.1007/s10071-009-0213-4 PMC2757608

[75] Therneau TM, Grambsch PM (2000) Modeling survival data: Extending the Cox model. New York: Springer.

[76] Therneau T (2013) A package for survival analysis in S. R package version 2.37-4.

[77] Team RC (2013) R: A language and environment for statistical computing. Vienna, Austria: R Foundation for Statistical Computing.

[78] Burnham KP, Anderson DR (2002) Model selection and multimodel inference: A practical information-theoretic approach. New York: Springer.

[79] Hoppitt W, Kandler A, Kendal JR, Laland KN (2010) The effect of task structure on diffusion dynamics: Implications for diffusion curve and network-based analyses. Learn Behav 38: 243–251. 20628163 10.3758/LB.38.3.243

