# Supplementary figures and images for: Social Network Analysis Shows Direct Evidence for Social Transmission of Tool Use in Wild Chimpanzees
Source: PLoS Biol. 2014 Sep 30;12(9):e1001960. doi: 10.1371/journal.pbio.1001960 (PMC4181963; doi:10.1371/journal.pbio.1001960)

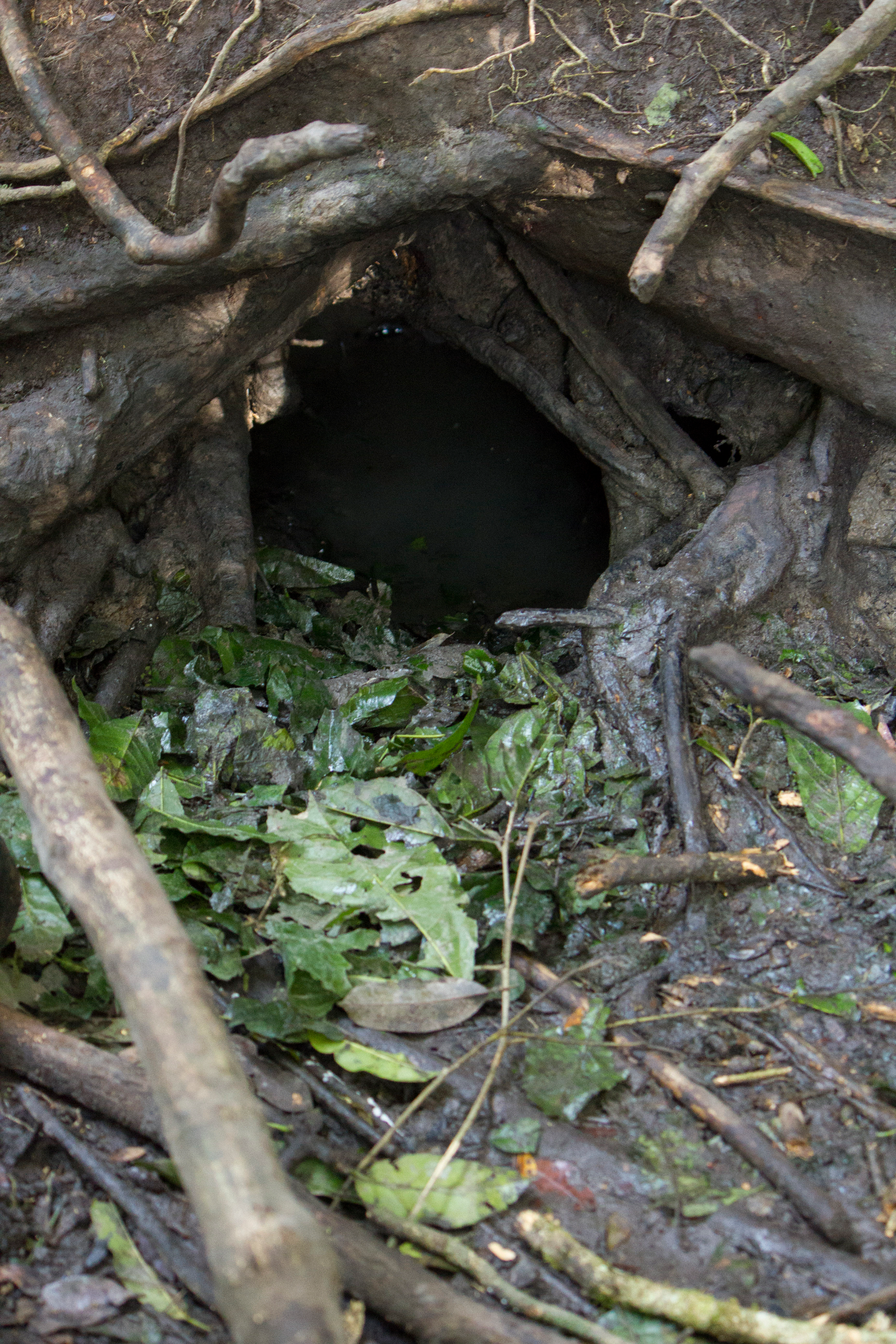

Supplement: Figure S1 — Location of the waterhole between the roots of the two trees (photo by Nina Hänninen, with permission). (TIF) [file pbio.1001960.s001.tif]

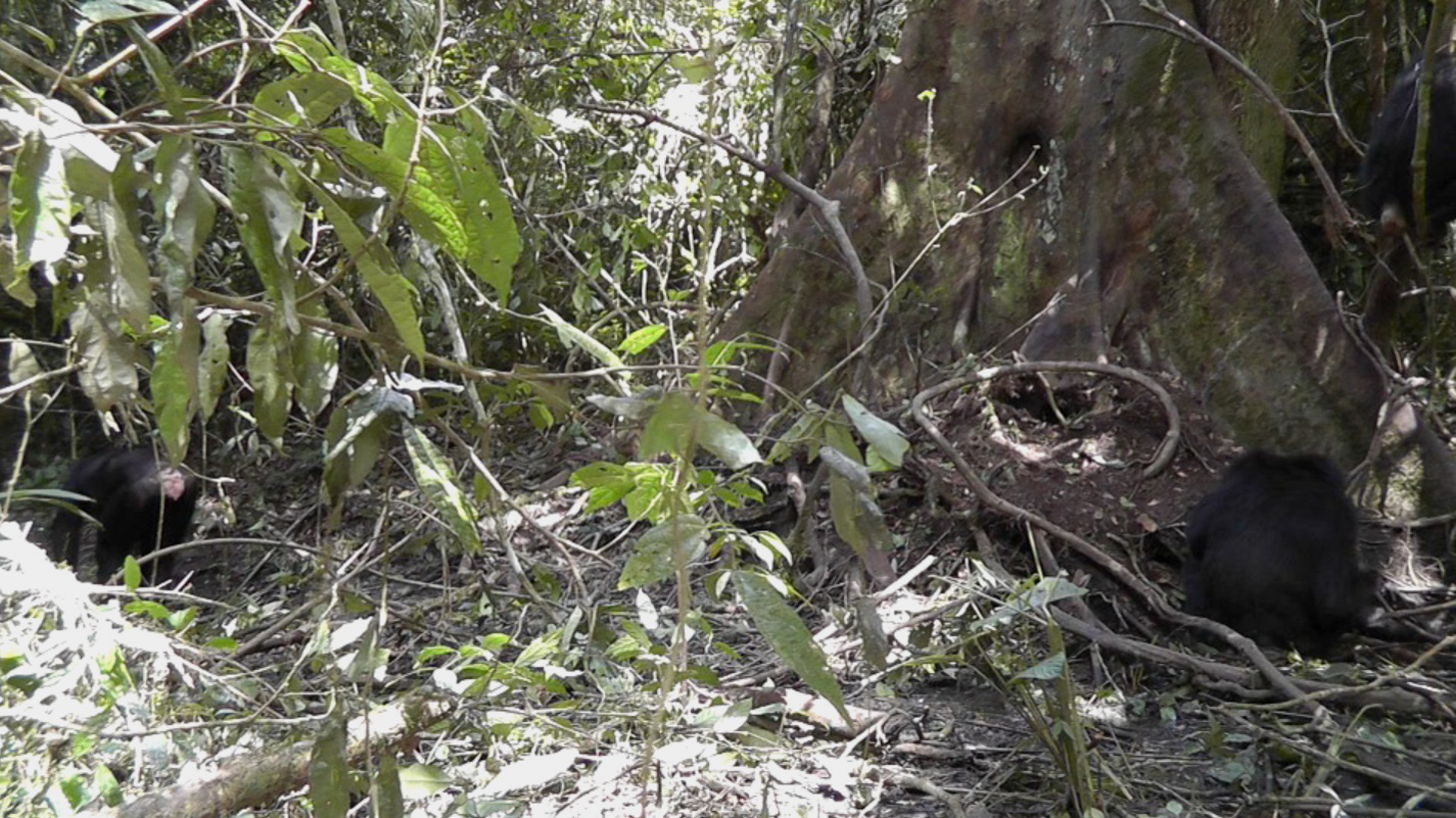

Supplement: Figure S2 — Broad view of the two trees (right, individual NB) and the puddles (left, individual OK) at the sponging location (photo by Catherine Hobaiter). (TIF) [file pbio.1001960.s002.tif]

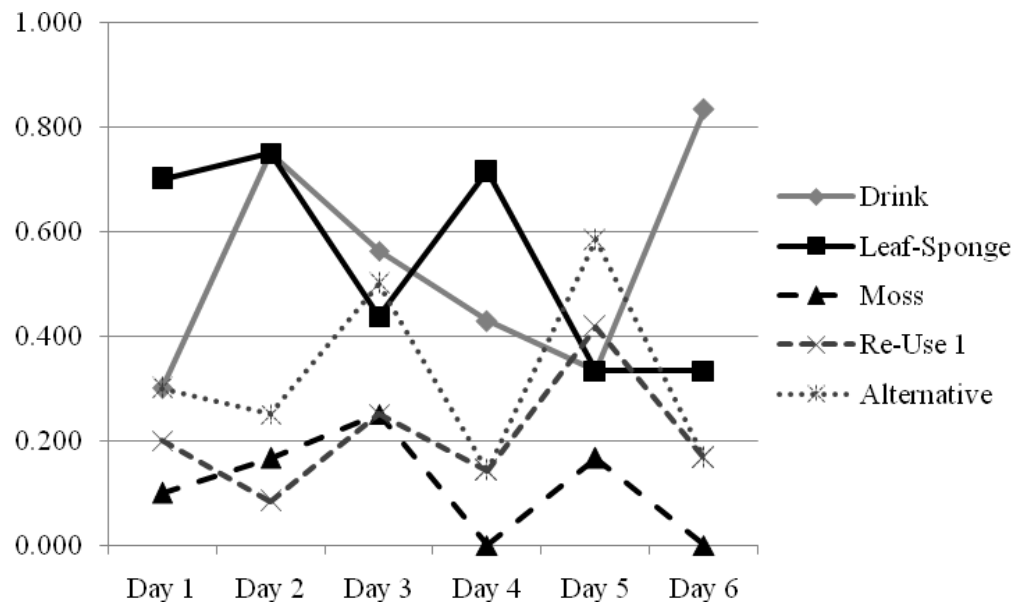

Supplement: Figure S3 — Proportion of individuals using different techniques at the waterhole (November 14–19). Drink, drink directly from the hole; Alternative, proportions of moss and re-use 1 combined. (PDF) [file pbio.1001960.s003.pdf]

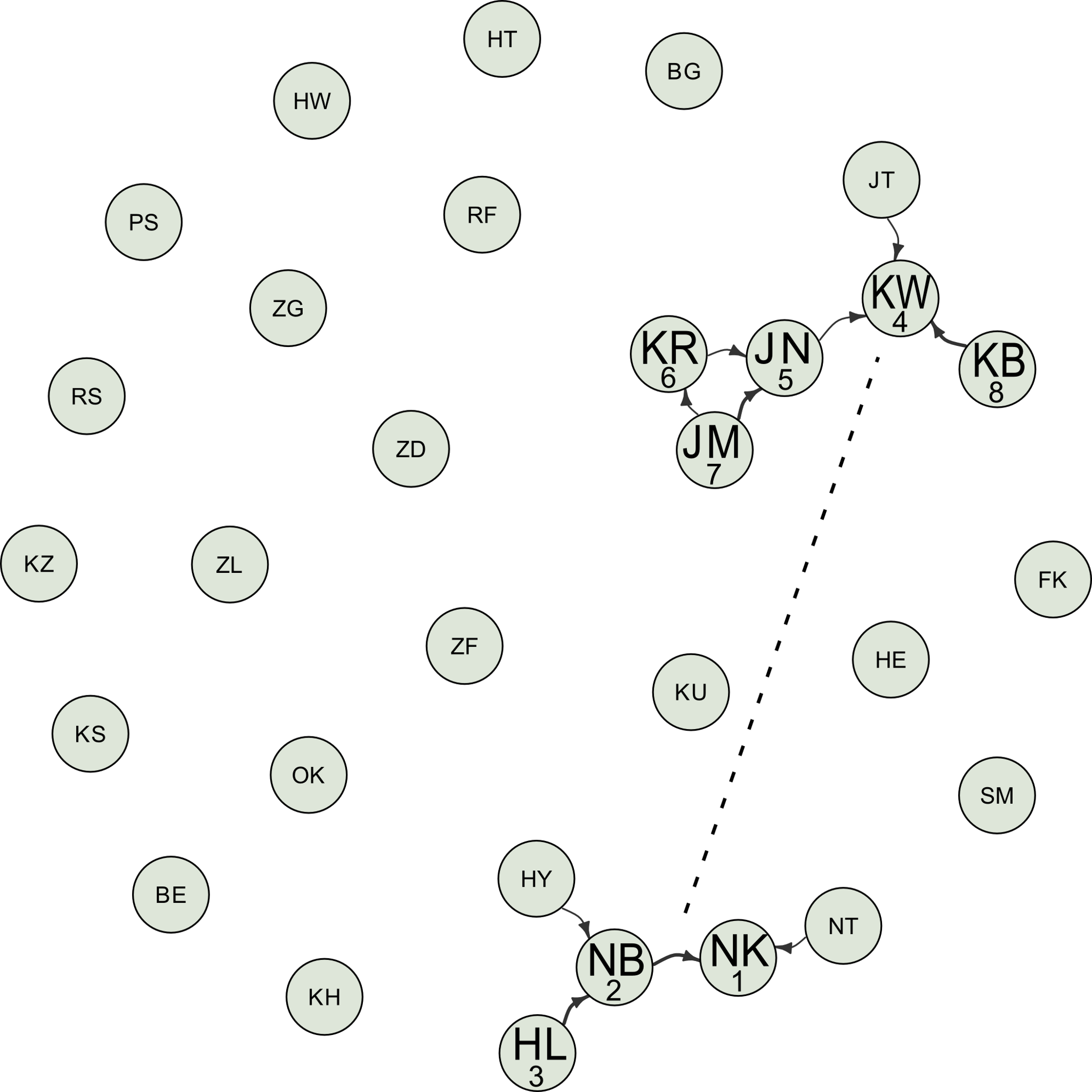

Supplement: Figure S4 — Visualization of the interaction networks for the moss-sponging behavior for all 30 individuals, in the case of the specific audience, using a stricter observation criterion (see Materials and Methods). Graphs are laid out using the Fruchterman–Reingold weighted algorithm. Labels on the nodes indicate the identity of individuals. Individuals with large label size developed the behavior, whereas individuals with small label size did not. Numbers under the large label indicate the order of acquisition of the behavior. The width of the arrows linking individuals is proportional to the number of times an interaction event was recorded between any two individuals and represented according to the convention “X→Y” means that Y was observed by X. Dashed line, potential product-based social learning by individual KW who re-used a moss-sponge. Data were deposited in the Dryad repository: http://dx.doi.org/10.5061/dryad.m6s21. (TIF) [file pbio.1001960.s004.tif]

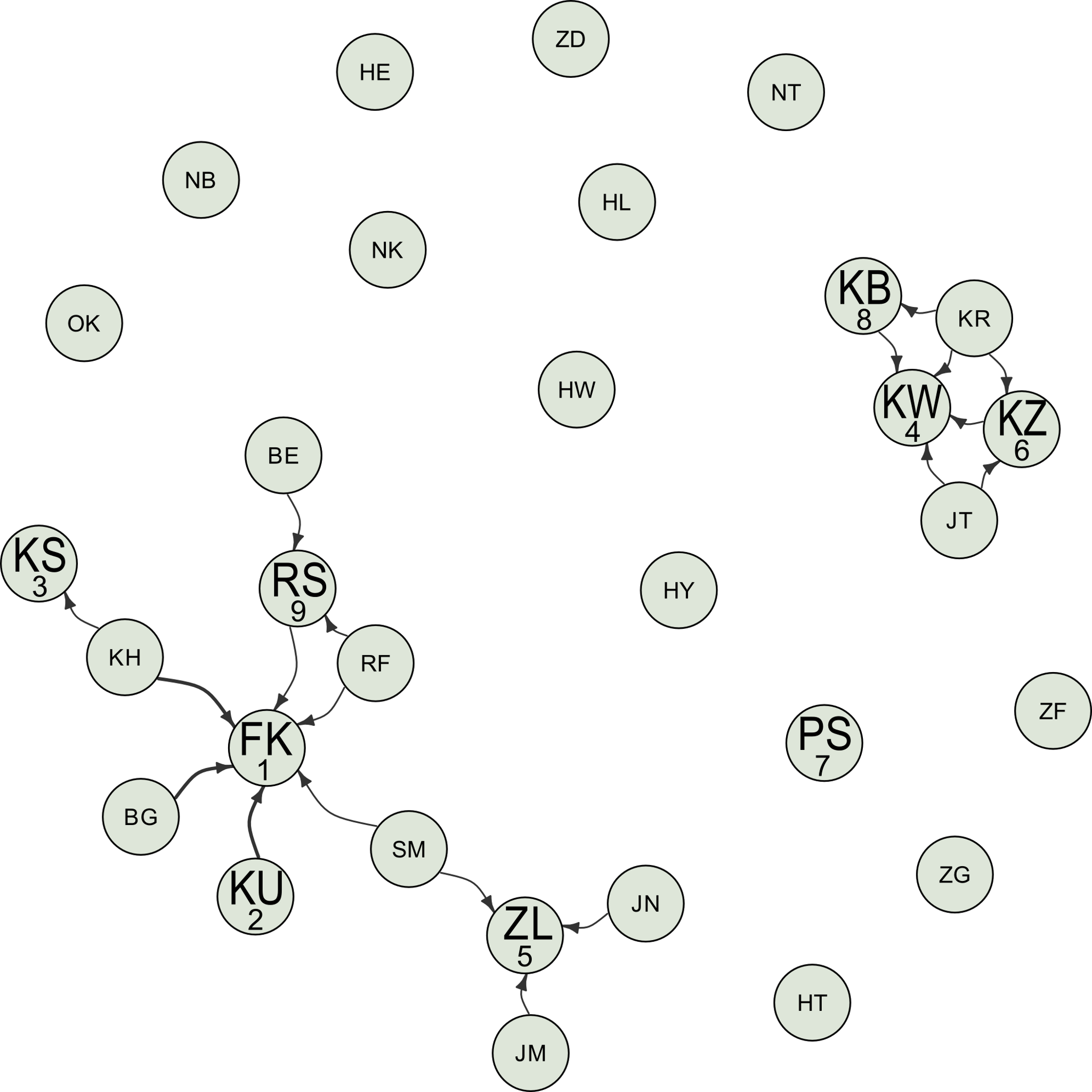

Supplement: Figure S5 — Visualization of the interaction networks for the RU1 behavior for all 30 individuals, in the case of the specific audience, using a stricter observation criterion (see Materials and Methods). Graphs are laid out using the Fruchterman–Reingold weighted algorithm. Labels on the nodes indicate the identity of individuals. Individuals with large label size developed the behavior, whereas individuals with small label size did not. Numbers under the large label indicate the order of acquisition of the behavior. The width of the arrows linking individuals is proportional to the number of times an interaction event was recorded between any two individuals and represented according to the convention “X→Y” means that Y was observed by X. Data were deposited in the Dryad repository: http://dx.doi.org/10.5061/dryad.m6s21. (TIF) [file pbio.1001960.s005.tif]

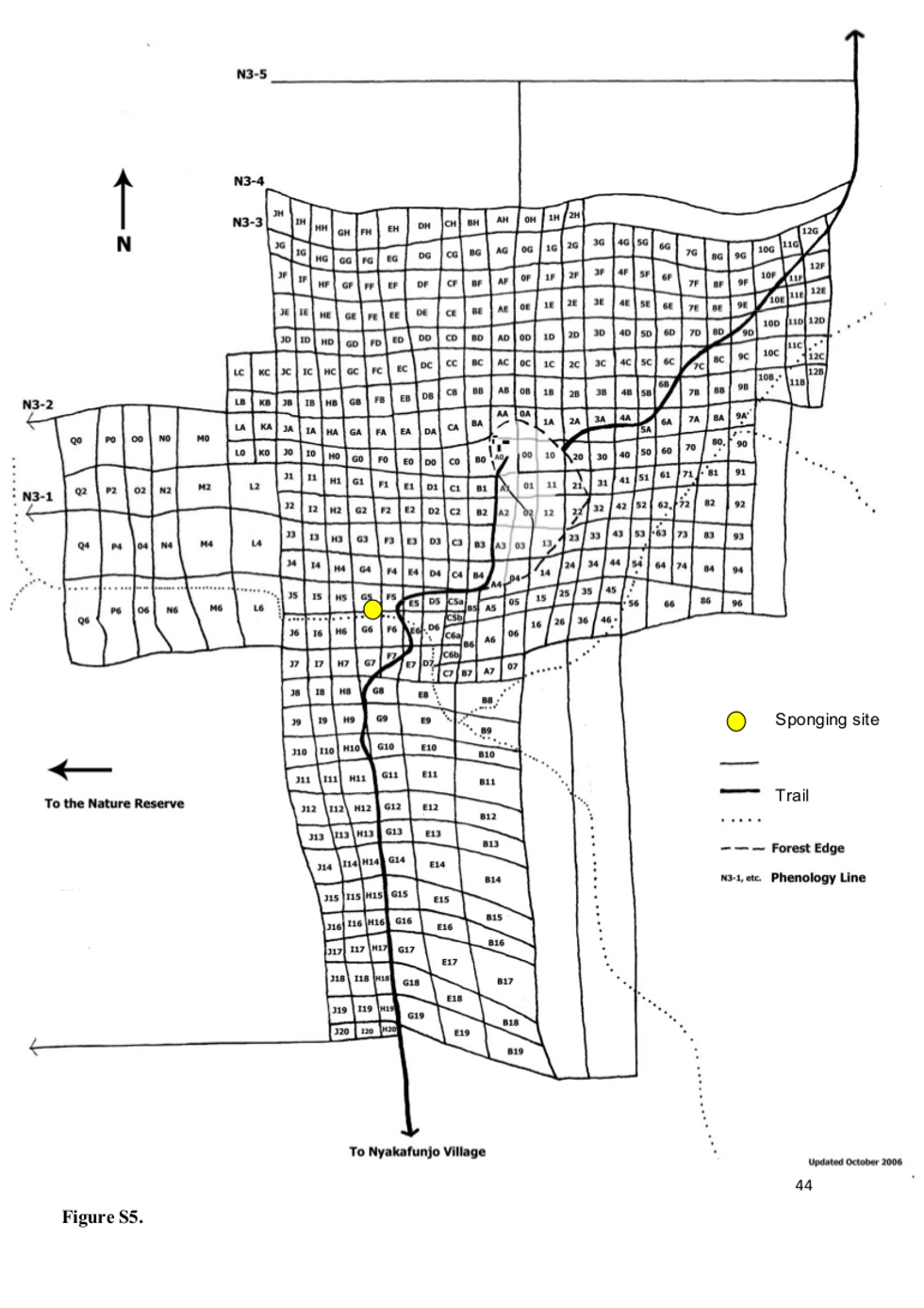

Supplement: Figure S7 — Location of the sponging site on the Budongo Conservation Field Station Grid System. (TIF) [file pbio.1001960.s007.tif]
