# Supplementary material for: Social Network Analysis Shows Direct Evidence for Social Transmission of Tool Use in Wild Chimpanzees
Source: PLoS Biol. 2014 Sep 30;12(9):e1001960. doi: 10.1371/journal.pbio.1001960 (PMC4181963; doi:10.1371/journal.pbio.1001960)
Supplement: Table S1 — Akaike weights for different social learning models assuming static or dynamic networks; with a linear, binary, or log-linear relationship with the rate of learning; with an additive or multiplicative interaction with individual level variables; and in which there was (a) equal social transmission for M and RU1, (b) differing levels of social transmission for M and RU1, (c) social transmission for M only, and (d) social transmission for RU1 only, compared with the Akaike weight for an asocial model. Akaike weights do not sum to 1 because a model with no individual-level variables qualifies as both an additive and multiplicative model. However, each cell represents the same number of models so the weights are directly comparable between cells. The upper panel corresponds to the analysis presented in the main text: Here the two cells with highest support account for 75% of the total support between them. Akaike weights are similar when KW's M acquisition event is excluded. The lower panel corresponds to the analysis based on the strict observation criteria and shows a similar pattern of results. (DOC) [file pbio.1001960.s008.doc]

| Network (Less strict) | Effect of increasing connections/ observations | Interaction with ILV’s | a) Same | b) Different | c) M only | d) RU1 only |
| --- | --- | --- | --- | --- | --- | --- |
| Static | Linear | Additive | 0.000 | 0.003 | 0.010 | 0.000 |
|  |  | Multiplicative | 0.000 | 0.003 | 0.014 | 0.000 |
| Dynamic | Linear | Additive | 0.008 | 0.023 | 0.018 | 0.000 |
|  |  | Multiplicative | 0.005 | 0.021 | 0.032 | 0.000 |
|  | Binary | Additive | 0.002 | 0.007 | 0.002 | 0.000 |
|  |  | Multiplicative | 0.001 | 0.008 | 0.002 | 0.000 |
|  | Log-Linear | Additive | 0.000 | 0.087 | 0.261 | 0.000 |
|  |  | Multiplicative | 0.000 | 0.160 | 0.490 | 0.000 |
| Asocial learning only | | | 0.000 |  |  |  |

| Network (strict) | Effect of increasing connections/ observations | Interaction with ILV’s | a) Same | b) Different | c) M only | d) RU1 only |
| --- | --- | --- | --- | --- | --- | --- |
| Static | Linear | Additive | 0.001 | 0.011 | 0.022 | 0.000 |
|  |  | Multiplicative | 0.003 | 0.012 | 0.048 | 0.000 |
| Dynamic | Linear | Additive | 0.035 | 0.086 | 0.036 | 0.000 |
|  |  | Multiplicative | 0.023 | 0.076 | 0.063 | 0.000 |
|  | Binary | Additive | 0.000 | 0.010 | 0.007 | 0.000 |
|  |  | Multiplicative | 0.007 | 0.007 | 0.000 | 0.000 |
|  | Log-Linear | Additive | 0.044 | 0.107 | 0.106 | 0.000 |
|  |  | Multiplicative | 0.028 | 0.205 | 0.219 | 0.000 |
| Asocial learning only | | | 0.000 |  |  |  |
