# Supplementary material for: Social Network Analysis Shows Direct Evidence for Social Transmission of Tool Use in Wild Chimpanzees
Source: PLoS Biol. 2014 Sep 30;12(9):e1001960. doi: 10.1371/journal.pbio.1001960 (PMC4181963; doi:10.1371/journal.pbio.1001960)
Supplement: Table S2 — Summary of results for individual-level variables, from the log-linear model using the dynamic network. Effects are given on the log scale with Wald confidence intervals calculated using the unconditional standard error. (DOC) [file pbio.1001960.s009.doc]

|  | Age (per year) | Sex (male- female) | Time (per year) |
| --- | --- | --- | --- |
| Total Akaike weight | 0.36 | 0.39 | 0.39 |
| Model-averaged estimate | 0.011 | 0.385 | 0.018 |
| 95% C.I. upper | 0.083 | 1.82 | 0.117 |
| 95% C.I. lower | -0.062 | -1.22 | -0.080 |
