# Supplementary material for: Social Network Analysis Shows Direct Evidence for Social Transmission of Tool Use in Wild Chimpanzees
Source: PLoS Biol. 2014 Sep 30;12(9):e1001960. doi: 10.1371/journal.pbio.1001960 (PMC4181963; doi:10.1371/journal.pbio.1001960)
Supplement: Table S3 — Pearson's correlation between techniques used by the chimpanzees and with days spent at the waterhole. LS, leaf-sponge; M, moss; RU1, re-use 1; ALT, alternative technique (M and RU1 combined); D, drink; DAY, days passed. To investigate whether the use of alternative techniques (M, RU1) was correlated to a decrease in available LS material, we ran Pearson's correlations using frequency of individual users per day per technique, and of each technique versus days passed, including drinking. If increased direct drinking were correlated with decreased users of LS, this may indicate an environmental constraint on tool production. There was no evidence of a correlation between the number of chimpanzees exhibiting the new techniques and the number of days passed (see Figure S3), suggesting that material availability did not influence tool choice. Furthermore, we found no correlations across days between the number of cases of LS and cases of either RU1, M, or RU1 and M combined, showing that selection of the techniques, old and new, were not associated. * In order to control for the varying number of individuals at the site per day, these tests are of number of individuals using the technique/total number of individuals, correlated against the number of days past. Degrees of freedom = 4 in all cases. All p values are two-tailed. (DOC) [file pbio.1001960.s010.doc]

| **Pairs** | **Pearson's r** | **P** |
| --- | --- | --- |
| LS - RU1 | -0.061 | 0.91 |
| LS – M | 0.527 | 0.28 |
| RU1 – M | 0.678 | 0.14 |
|  |  |  |
| LS – ALT | 0.231 | 0.66 |
|  |  |  |
| D* - DAY | 0.306 | 0.56 |
| LS* - DAY | -0.765 | 0.08 |
| RU1* - DAY | 0.345 | 0.50 |
| M* - DAY | -0.397 | 0.44 |
| ALT* - DAY | -0.006 | 0.99 |
