# Supplementary material for: Social Network Analysis Shows Direct Evidence for Social Transmission of Tool Use in Wild Chimpanzees
Source: PLoS Biol. 2014 Sep 30;12(9):e1001960. doi: 10.1371/journal.pbio.1001960 (PMC4181963; doi:10.1371/journal.pbio.1001960)
Supplement: Table S4 — List of Sonso individuals who manufactured at least one leaf-based tool at the waterhole in the course of the 6 d with individual information as of November 2011. Individual identity code, age (expressed in years), sex (F, female; M, male), age class, family (code of the mother), and tenure (time spent within the community expressed in years) are provided. Note that age and tenure estimates for individuals over 20 y are estimates and should be treated as ±3 y. (DOC) [file pbio.1001960.s011.doc]

| ID | Age | Sex | Age class | Family | Time spent in community as of 2011 |
| --- | --- | --- | --- | --- | --- |
| BE | 35 | F | Adult | BE | 7 |
| BG | 7 | F | Juvenile | BE | 7 |
| FK | 12 | M | Sub-Adult | FL | 12 |
| HE | 2 | F | Infant | HT | 2 |
| HL | 10 | F | Sub-Adult | HT | 10 |
| HT | 33 | F | Adult | HT | 18 |
| HW | 18 | M | Adult | HT | 18 |
| HY | 6 | F | Juvenile | HT | 6 |
| JM | 5 | M | Juvenile | JN | 5 |
| JN | 27 | F | Adult | JN | 16 |
| JT | 12 | F | Sub-Adult | JN | 12 |
| KB | 4 | F | Infant | KW | 4 |
| KH | 3 | F | Infant | KU | 3 |
| KR | 10 | F | Sub-Adult | KW | 10 |
| KS | 8 | M | Juvenile | KU | 8 |
| KU | 32 | F | Adult | KU | 19 |
| KW | 30 | F | Adult | KW | 19 |
| KZ | 16 | M | Adult | KW | 16 |
| NK | 29 | M | Adult | RH | 29 |
| NB | 49 | F | Adult | NB | 34 |
| NT | 8 | F | Juvenile | NB | 8 |
| OK | 15 | F | Adult | OK | 1 |
| PS | 13 | M | Sub-Adult | PL | 13 |
| RF | 4 | F | Infant | RH | 4 |
| RS | 14 | F | Sub-Adult | RH | 14 |
| SM | 18 | M | Adult | SE | 18 |
| ZD | 10 | M | Sub-Adult | ZN | 10 |
| ZF | 29 | M | Adult | BN | 29 |
| ZG | 14 | M | Sub-Adult | ZM | 14 |
| ZL | 16 | M | Adult | ZN | 16 |
